# Depression symptoms and suicidal ideation among HIV infected Rwandans: the mediating and moderating effects of complicated grief and substance abuse

**DOI:** 10.1186/s12981-024-00628-1

**Published:** 2024-06-07

**Authors:** Anualitha Uwiringiyimana, Japhet Niyonsenga, Kethina Gaju Lisette, Athanasie Bugenimana, Jean Mutabaruka, Augustin Nshimiyimana

**Affiliations:** 1 Department of Clinical Psychology, College of Medicine and Health Sciences, University of Rwanda, Kigali, Rwanda; 2 Mental Health and Behaviour Research Group, College of Medicine and Health Sciences, University of Rwanda, Kigali, Rwanda; 3 Department of Global Mental Health, London Kings College, London, UK; 4 Department of General Medicine and Surgery, College of Medicine and Health Sciences, University of Rwanda, Kigali, Rwanda

**Keywords:** Depression symptoms, Complicated grief, Substance abuse, Suicidal ideation, HIV infected person

## Abstract

**Background:**

People with HIV/AIDS (PWHA) have 7–36 times greater risk for completed suicide associated with depression symptoms compared to general population. However, no study has sufficiently analyzed the mediating or moderating variables of the relationship between depression and suicidal ideation in Rwanda.

**Objectives:**

This study aimed to examine how complicated grief mediates and substance abuse moderates the effects of depression symptoms on suicidal ideation.

**Methods:**

Data were collected from a convenient sample of 140 participants (M-age = 38.79 years, SD = 10.218) receiving antiretroviral therapy (ART) at Remera Health Center in a cross-sectional study. Multiple linear regression and Sobel test were used to examine the relationships between depression symptoms, complicated grief, suicidal ideation, and substance abuse.

**Results:**

The results indicated that 29% of the sample had clinically significant symptoms of depression and 18% had suicidal ideation. The interaction between substance abuse and depression symptoms (β = .468, t = 8.02, p = 0.000) was a significant predictor, explaining the 55.7% of variance in suicidal ideation. Furthermore, the Sobel test demonstrated that complicated grief mediated the effects of depression symptoms (t = 4.67, *SE* = 0.0101, p ≤ 0.001) on suicidal ideation.

**Conclusion:**

The results suggest that depression symptoms are associated with an increased risk of suicidal ideation, and this risk significantly amplified in the presence of complicated grief and substance abuse. These findings highlight the importance of integrating mental health services, particularly those addressing depression, complicated grief, and substance abuse, into HIV care programs to mitigate the risk of suicidal ideation among PWHA.

## Introduction

HIV infection is a significant global public health problem, with about 38 million people living with HIV, one million deaths from related causes in 2019, and an estimated 1.7 million people new infections expected in the following years [16, 49]. The disease has led to 32.7 million deaths globally since the 1980s–2019 [49]. Sub-Saharan Africa is the most affected region, accounting for more than 66% of new HIV infections [16]. Within this region, countries in southern and eastern Africa, including Rwanda are the epicenters of the pandemic, with the highest prevalence of HIV reported in South Africa (17.9%), Botswana (23.4%) and Swaziland (24.8%), [16]. In Rwanda, the prevalence of HIV among adults aged 15–49 years and those aged 15–64 years was 2.6% and 3%, respectively in 2018–2019, with 5400 new cases of HIV per year [33]. Notably, HIV and depressive disorders are projected to be the top two leading causes of burden of disease globally by 2030 [26].

Depression is the most common mental disorder reported in individuals with HIV/AIDS (PWHA), second only to substance abuse [43]. Depression is two to three-times more common in people living with HIV (PLWH) than in the general population [9, 43]. Additionally, the co-occurrence of depression symptoms, substance abuse [6], suicidal ideation [18], and complicated grief [7] among PWHA is well documented. Alarmingly, PWHA have 7–36 times greater risk for completed suicide compared to the general population [18]. Suicide is a major global health burden, causing 800,000 deaths per year worldwide, with 78% of these occurring in low-and middle-income countries [51]. In Rwanda, suicide causes about 12 per 100,000 deaths per year [52]. For every single completed suicide, there are about 20 suicide attempts [52], indicating a higher-level suicidal thought (ideation).

While depression and suicidal ideation are closely related, they represent distinct constructs. Suicidal ideation warrants separate examination due to its unique implications for prevention and intervention strategies [32]. This distinction is important for understanding the specific risk factors and mechanisms that contribute to suicidal ideation independently of general depressive symptoms [19]. Depressive symptoms often involve hopelessness and low mood, while suicidal ideation involves thoughts about self-harm or ending one’s life, arising from different cognitive and emotional processes [13]. Focusing specifically on suicidal ideation provides insight into unique aspects that are critical for targeted interventions and effective prevention efforts. Research shows that factors such as impulsivity, burdensomeness, and lack of belongingness are more directly related to suicidal ideation than depression itself [50]. By identifying these distinct factors, mental health professionals can develop targeted strategies to address and mitigate the risk of suicide. Furthermore, understanding the unique pathways leading to suicidal ideation can help in creating specialized therapeutic approaches and improving screening tools to better identify individuals at risk [11]. Therefore, differentiating between depression and suicidal ideation in research and practice is essential for effective prevention and intervention strategies.

Compelling evidence shows that depressive symptoms, complicated grief, substance abuse and suicidal ideation often co-occur among PLWHA, potentially due to the direct impact of HIV infection or the side effect of medication [6, 7, 18]. However, little is known about how these variables influence each other. This study aims to examine how complicated grief mediates and substance abuse moderates the effects of depression symptoms on suicidal ideation among PLWHA.

Clarifying the relationship between depression and grief is essential, as they are distinct yet overlapping constructs. Depression involves persistent low mood and loss of interest, while grief encompasses a broad range of emotions in response to loss, including sadness and anger [39, 53]. While depression can be part of grief, it is not universal, and grief can lead to complicated grief, characterized by prolonged, intense symptoms that interfere with daily functioning [44]. Understanding these distinctions is crucial for developing effective interventions and supports tailored to the specific needs of individuals experiencing grief versus those with depression. Suicidal ideation in grief may stem from a desire to reunite with the deceased, whereas in depression, it may arise from feelings of hopelessness and worthlessness. Recognizing these differences helps mental health professionals better identify underlying causes of suicidal ideation and develop targeted prevention strategies [14].

Literature highlights that complicated grief, characterized by persistent grief symptoms distinct from normal bereavement-related depression and anxiety, is a robust predictor of suicidal ideation [27]. In a study by Szanto et al. [47], investigating the link between complicated grief and suicidal ideation among 130 elderly who had lost their spouses, participants with high scores on complicated grief reported higher suicidal ideation than those with low scores (57% versus 24%) during the follow up period. Similarly, Latham and Prigerson [23] found that the risk of suicidal thoughts or behaviors is 10.80 times greater for individuals with complicated grief and 7.10 times greater for those with a major depressive disorder compared to those without these disorders. Therefore, we hypothesize that complicated grief mediates the effects of depression on suicidal ideation.

Substance abuse may moderate the relationship of depression symptoms and suicidal ideation through self-medication processes. Recent studies have shown that the presence of depression symptoms and substance abuse in individuals with HIV/AIDS increases the risk of suicide [34]. Substance abuse, used as a means to alleviate psychological problems, impacts the psychological, environmental, and social factors that make suicide more likely [37]. Greater severity of alcohol and drugs use is associated with an elevated risk of suicide attempts and mortality [29]. Co-occurring alcohol and drug use disorders and HIV/AIDS are strong indicators of increased suicide risk. Thus, we hypothesize that substance abuse moderates the effects of depression symptoms on suicidal ideation among PWHA.

Overall, the main objectives of this study are two-fold: to examine whether complicated grief mediates the effects of depression on suicidal ideation, and to examine whether substance abuse moderates the effects of depression symptoms on suicidal ideation. We hypothesize that the effects of depression on suicidal ideation are mediated by complicated grief, and moderated by substance abuse among PLWHA.

## Methods

### Participants

The selection of HIV positive patients was based on a convenience sampling method. A total of 140 HIV/AIDS positive patients (females 70.6%, mean age = 38.79, SD = 10.218) receiving antiretroviral therapy (ART) at Remera health center participated in this cross-sectional study. The inclusion criteria incorporated the willingness to participate in the study, being HIV/AIDS positive and being aged 18 and above.

### Instruments

All measures were first translated into Kinyarwanda by a team of three clinical psychologists and then back-translated by another team of four clinical psychologists all speaking English and Kinyarwanda. A self-designed questionnaire was used to record socio-demographic characteristics (age, sex, marital status, education, and monthly income). All tools demonstrated suitable level of reliability, with Cronbach’s alpha values ranging from 0.70 to 0.925.

### The inventory of complicated grief revised (ICG-R)

The Inventory of Complicated Grief Revised (ICG-R): This instrument consists of 19 items scored on a 5-point Likert scale ranging from 0 (never) to 4 (very often), indicating an increasing severity [40]. The cut-off point of ICG-R was ˃25, indicating the presence of Complicated Grief. The internal consistency of ICG-R in the sample was high (Cronbach’s alpha = 0.925).

### The suicide behaviors questionnaire-revised (SBQ-R)

“The Suicide Behaviors Questionnaire-revised (SBQ-R): This instrument consists of 4 questions accessing suicidality across four dimensions [35]. For the current study, we used only Item 2 (“How often have you thought about killing yourself over the past year?”), which assesses the frequency of recent suicidal ideation. The item was scored on a five-point scale, ranging from 1 (Never) to 5 (Very often).

### The alcohol, smoking and substance involvement screening test (ASSIST)

“The Alcohol, Smoking and Substance Involvement Screening Test (ASSIST)”: This 28-item measure that uses a five-point Likert scale, ranging from 1 (Almost Never) to 5 (almost always) to assess alcohol and other substance abuse [12]. The cut-off scores of ≥ 50 indicate the presence of alcohol and other substance abuse among participants. The Cronbach’s alpha in the sample was 0.920.

### Adult severity measure of depression adapted from the patient health questionnaire-9 (PHQ-9)

“Adult Severity Measure of Depression adapted from the Patient Health Questionnaire-9 (PHQ-9)”: This instrument consists of 9 items scored on a four-point Likert scale, ranging from 0 (Not at all) to 3 (Nearly every day), to quickly screen depressive symptomatology in adults over age 18 [21]. The cut-off point on this scale was ≥ 10, with a Cronbach’s alpha of 0.809 in the sample.

### Procedure

The study was conducted at Remera health centre in Gasabo district. Participants were selected based on the inclusion criteria. All participants were first informed about the study’s purpose, procedures, confidentiality terms and their right to withdraw at any time. No incentives or other motivations were provided for participation. Informed consent was obtained from all participants before starting the interview. After appreciation of the College of Medicine and Health Sciences institutional review board, the study protocol was approved by the committee of the Clinical Psychology Department prior to proceed with the data collection. All interviews were conducted by a trained clinical psychologist.

### Data analysis

Data analysis was conducted using the Statistical Package for Social Sciences (SPSS) version 28.0 software for Windows. Pearson’s correlation coefficient (r) was used to identify correlations between variables. Additionally, hierarchical multiple linear regressions were performed to determine the factors of suicidal ideation among HIV positive patients. A multiple linear regression analysis was conducted to examine the moderating effects of substance abuse, while the mediating effects of complicated grief were evaluated using the three step approach recommended by Baron and Kenny [1] and Judd, Kenny, and McClelland [15] along with Sobel tests [46] to further test the statistical significance of the mediation. Before performing linear regression analysis, four core assumptions of this analysis were given full consideration. After removing significant outliers, normal distribution, homoscedasticity, and multicollinearity were verified. The predictor variables had tolerance values ≥ 0.37, eliminating multicollinearity concerns. To determine the strength of association between dependent and independent variables, we used a 95% confidence level and a p-value of less than 0.05 to test hypotheses.

## Results

### Socio-demographic characteristics

The majority of the participant were aged between 30 and 50 years (M_age = 38.79, SD = 10.218). The sample was predominantly female (n = 99, 70.6%) and most participants were married (n = 57, 40.5%). In terms of education, the highest number of participants had completed the primary (n = 54, 38.6%), followed by high school (n = 37, 26.4%), illiterate (n = 28, 20%), vocation training (n = 12, 8.6%) and university education (n = 9, 6.4%) (Table 1).

**Table 1:** socio-demographics characteristics

Variables	Category	Frequency	Percentages
1. Gender			
	Male	41	29.4%
	Female	99	70.6%
2. Marital status			
	Single	34	24.6%
	Widow	28	19.8%
	Married	57	40.5%
	Divorced	15	11.1%
	Others	4	4%
3. Education			
	Primary	54	38.6%
	High school	37	26.4%
	Illiterate	28	20%
	Vocation	12	8.6%
	University	9	6.4%

### Descriptive statistics and correlation between variables

As demonstrated in Table 2, there were significant correlations of suicidal ideation with depression symptoms, complicated grief, age, and substance abuse. Additionally, there was also a notable inter-correlation between variables. However, gender and marital status were not associated with suicidal ideation.

**Table 2:** Descriptive statistics and Inter-correlations between variables

	Mean	SD	PHQ-9	CG	SI	ASSIST	MI	Age
Depression (PHQ-9)	5.76	4.40	1	0.557^**^	0.612^**^	0.503^**^	− 0.145	− 0.233^**^
Complicated Grief (CG)	23.00	15.51		1	0.447^**^	0.383^**^	− 0.117	− 0.18^*^
Suicidal ideation (SI)	0.642	1.31			1	0.510^**^	− 0.117	− 0.31^**^
Substance abuse (ASSIST)	40.48	14.96				1	− 0.091	− 0.27^**^
Monthly income (MI)	36214	48393.6					1	0.119
Age	38.78	10.22						1

^**^p ≤ 0.001 and ^*^ p ≤ *0.05*

### Factors of suicidal ideation among HIV positive patients

To determine the factors of suicidal ideation among HIV positive patients, we performed hierarchical multiple regression analysis in two steps. Step1: A model was composed all independent variables and only depression symptoms (β = 0.418, t = 4.816, p < 0.001), age (β = − 0.21, t = − 2.82, p = 0.010), substance abuse (β = 0.2, t = 2.6, p = 002), and marital status (β = 0.18, t = 2.34, p = 0.03) predicted suicidal ideation. However, complicated grief (β = 0.117, t = 1.420, p = 0.158) and gender (β = − 0.064, t = − 0.910, p = 0.365) were not significant predictors of suicidal ideation. In Step 2, after excluding depression symptoms from the model, and the results showed that Substance abuse (β = 0.327, t = 3.92, p < 0.001) and complicated grief (β = 0.292, t = 3.639, p < 0.001), and age(β = − 0.218, t = − 2.640, p = 0.009) predicted suicidal ideation, whereas marital status (β = 0.132, t = 1.692, p = 0.93) and gender (β = − 0.062, t = − 0.803, p = 0.424) did not.

### The mediating effects of complicated grief between depressive symptoms and suicidal ideation

The mediation models were examined by following the three steps suggested by Baron and Kenny [1] and Judd, Kenny, and McClelland [15]: (1) The effects of depression symptoms (B = 0.118, β = 0.612, t = 8.620, p < 0.001, R^2^ = 0.375) on suicidal ideation were statistically significant; (2) the effects of depression symptoms (B = 1.963, β = 0.557, t = 7.471, p < 0.001, R^2^ = 0.310) on complicated grief were statistically significant; and (3) the effects of complicated grief (B = 0.024, β = 0.447, t = 5.558, p < 0.001, R^2^ = 0.199) on suicidal ideation were statistically significant. As these preliminary conditions indicated that complicated grief mediated the effects of depression symptoms on suicidal ideation, we performed Sobel tests [46] to test the statistical significance of the mediation. The Sobel test results demonstrated that complicated grief mediated effects of depression symptoms (t = 4.67, *SE* = 0.0101, p ≤ 0.001) on suicidal ideation among HIV positive patients.

### Moderating effects of substance abuse on the relationships between depression symptoms and suicidal ideation among PWHA

**Fig. 1:**
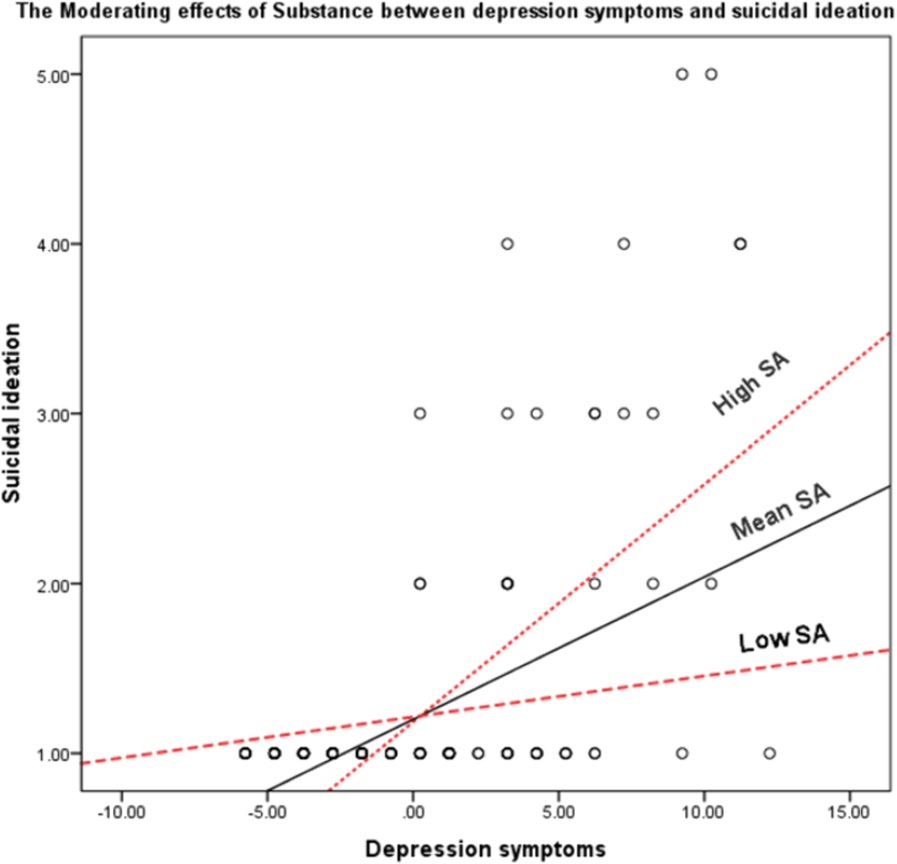
The moderating effects of substance abuse (SA) between depression symptoms and suicidal ideation

Multiple linear regression analysis was used to determine the moderating effects of substance abuse on the relationship between depression symptoms and suicidal ideation among HIV positive patients. Results indicated that the interaction between substance abuse and depression symptoms (B = 0.004, β = 0.468, t = 8.02, p = 0.000) was a significant predictor, explaining the 55.7% of variance in suicidal ideation (Fig. 1).

## Discussion

To the best of our knowledge, this study is the first to report the association of depression symptoms, complicated grief, and substance abuse with suicidal ideation (SI) in terms of mediation and moderation among HIV positive patients from low-income country with history of Genocide, where information on suicide is limited. Worryingly, the evidence shows that 75% of the global suicides take place in low and middle-income countries [45]. Rwanda has exception as the HIV/AIDS and mental disorders prevalence has been affected the 1994 Tutsi genocide, during which nearly one-seventh Rwandans were killed, 250 000 women were raped, and millions of Rwandans were displaced [2, 36]. As hypothesized and supported by prior studies, depression symptoms [30, 34, 42], substance abuse [5, 6, 38], complicated grief [3, 24, 47, 48] and age [45] was associated with suicidal ideations (SI).

As hypothesized and supported by prior studies, depressive symptoms [34, 34, 42], substance abuse [5, 6, 38], complicated grief [3, 24, 47, 48], and age [45] are associated with suicidal ideation. Depressive symptoms may contribute to suicidal ideation through feelings of hopelessness and worthlessness, cognitive distortions, and pervasive despair [13]. Substance abuse increases risk by impairing judgment, reducing inhibitions, and serving as a maladaptive coping mechanism, exacerbating emotional distress [10]. Complicated grief leads to intense emotional pain and functional impairment, making suicide seem like an escape from suffering [44]. Age-related factors also play a role, with older adults facing issues like social isolation and chronic illness [4], while younger individuals may struggle with academic, social, and identity pressures [17]. Understanding these associations highlights the need for comprehensive mental health care that addresses these multifaceted factors to mitigate the risk of suicide.

Our findings indicate that complicated grief mediates the effects of depression symptoms on suicidal ideation among HIV-positive patients. This mediation effect aligns with existing literature that suggests a strong inter-correlation and frequent co-occurrence of depression, complicated grief, and suicidal thoughts [3, 24, 34, 48]. Latham and Prigerson [23] found that the risk of suicidal thoughts was approximately seven times higher for individuals with ‘complicated grief’ and slightly over seven times higher for those with ‘major depressive disorder’. Further, a meta-analysis of psychological disorders indicated that 41% of bereaved adults showed clinically significant symptoms of depression [20]. Although only a small proportion of bereaved individuals experience complicated grief that causes persistent suffering [22, 41], almost 10% of bereaved adults report symptoms of complicated grief after non-traumatic loss (i.e., loss due to old age or sickness; [25]), with this proportion increasing to 49% after traumatic loss (i.e., loss due to murder, terror, or natural disasters; [8]).

Our study also highlights that substance abuse moderates the relationship between depression symptoms and suicidal ideation. This finding supports the notion that substance abuse may may serve as self-medication process. For HIV-infected person to cope with stressful and disturbing symptoms of depression [31]. Previous studies have also reported that presence of depression symptoms and substance abuse in individuals HIV/AIDS with may increase the risk of suicide [34]. Additionally, substance abuse exacerbates both symptoms of depression [31] and suicide ideation [5, 38]. For alcohol abuse, a high severity of recent drinking is associated with a greater likelihood of suicide attempts and mortality [29]. Co-occurring substance use disorders and depression symptoms are salient indicators of elevated risk of suicide among HIV-infected individuals.

## Limitation

The limitations of this study include the use of convenience sampling, which does not allow the researcher to confirm the sample as representative of the overall population of PWHA due to the lack of randomization [28]. Consequently, generalizability of this study is constrained by the sample. Additionally, the cross-sectional study design relies on self-report surveys which are subject to respondent bias, where participants may present themselves in a more positive light than reality [28]. To reduce this possibility of error, random presentation of the instruments was applied, and namelessness was guaranteed to also reduce the social desirability bias. Future research should consider using larger sample sizes to explore the potential moderated mediation effects of substance abuse on the relationship between depressive symptoms, complicated grief, and suicidal ideation.

## Conclusion

Results of this study highlight that depression symptoms, complicated grief and substance abuse predicted suicidal ideation among HIV/AIDS positive patients. Moreover, substance abuse moderates the effects of depression symptoms on suicidal ideation, while complicated grief mediates the relationship between depression symptoms and suicidal ideation. These findings may be valuable to health care providers, family, friends, community, and policymakers in supporting HIV/AIDS positive patients who are contemplating suicide. To reduce the incidence of suicidal ideation among PWHA, routine screenings for substance abuse, depression symptoms and complicated grief in HIV/AIDS positive patients are recommended for health care providers to consider in further management.

## Data Availability

The datasets used and/or analysed during the current study are available from the corresponding author on reasonable request.
